# Suppression of ferroptosis through the SLC7A11/glutathione/glutathione peroxidase 4 axis contributes to the therapeutic action of the Tangshenning formula on diabetic renal tubular injury

**DOI:** 10.1186/s13020-024-01007-8

**Published:** 2024-10-29

**Authors:** Xiao-Meng Shan, Chun-Wei Chen, Da-Wei Zou, Yan-Bin Gao, Yin-Ying Ba, Jia-Xin He, Zhi-Yao Zhu, Jia-Jun Liang

**Affiliations:** 1 School of Traditional Chinese Medicine, Capital Medical University, #10, Youanmenwai, Xitoutiao, Fengtai District, Beijing, 100069 People’s Republic of China; 2 Beijing Key Lab of TCM Collateral Disease Theory Research, #10, Youanmenwai, Xitoutiao, Fengtai District, Beijing, 100069 People’s Republic of China

**Keywords:** Diabetic nephropathy, Tangshenning formula, Renal tubular injury, Ferroptosis, SLC7A11

## Abstract

**Background:**

Tangshenning (TSN) is a safe and effective formula to treat diabetic nephropathy (DN), and clinical studies have demonstrated that its therapeutic effects are related to oxidative stress improvements in patients. Herein, this study aims to explore the potential mechanism of how TSN alleviates diabetic renal tubular injury.

**Methods:**

The ultrahigh pressure liquid chromatography-quadrupole-time of flight mass spectrometry (UPLC-QTOF/MS) was used to identify the chemical composition and serum components of TSN. KK-Ay mice served to investigate the protective effects and regulatory mechanisms of TSN on tubular damage in DN. Furthermore, inhibitors and inducers of ferroptosis were employed in high glucose-cultured tubular epithelial cells (TECs) to verify the potential mechanisms of TSN. The expressions of proteins related to renal tubular injury, ferroptosis and solute carrier family 7, member 11 (SLC7A11)/glutathione (GSH)/glutathione peroxidase 4 (GPX4) axis were analyzed by western blot and immunofluorescence. Mitochondrial ultrastructure was observed in kidney tissues and TECs by a transmission electron microscope. Pathological changes in the renal tissues were observed by HE, PAS, and Prussian blue staining. Ferroptosis-related reactive oxygen species (ROS), malondialdehyde (MDA), ferrous ion, the intake of cystine, GSH, and oxidized glutathione (GSSG) were evaluated and contrasted in vivo or in vitro.

**Results:**

51 compounds of TSN powder and 11 components in TSN-containing serum were identified by UPLC-QTOF/MS method. Administration of TSN ameliorated the elevated levels of proteinuria, serum creatinine, blood urea nitrogen, abnormal expression of renal tubular injury markers, and pathological damage to the renal tubules in DN mice model. Intriguingly, a strong inhibition of ferroptosis after TSN treatment occurred in both DN mice model and high glucose-cultured TECs. Notably, induction of ferroptosis by erastin attenuated the protective effect of TSN in high glucose-cultured TECs, while the ferroptosis inhibition by ferrostatin-1 treatment protected renal tubular, which was similar to TSN, suggesting the contribution of TSN-mediated by the inhibition of ferroptosis in DN progression. Mechanistically, TSN upregulated the SLC7A11/GSH/GPX4 axis to inhibit ferroptosis.

**Conclusion:**

TSN may delay the DN progression and attenuate the renal tubular injury by inhibiting the ferroptosis regulated by the SLC7A11/GSH/GPX4 axis.

**Supplementary Information:**

The online version contains supplementary material available at 10.1186/s13020-024-01007-8.

## Introduction

The global prevalence rate of diabetes mellitus is expected to increase by 12.2% in 2045 [1]. More dreadfully, renal failure caused by diabetic nephropathy (DN) is regarded as the primary reason for renal replacement therapy worldwide [1, 2]. So far, the treatment for DN has mainly focused on reducing blood glucose levels, lowering blood lipids, managing blood pressure, and implementing dietary therapy. Moreover, angiotensin-converting enzyme inhibitors (ACEIs) or angiotensin receptor blockers (ARBs) are applied as the first-line medications. However, these treatment options have limited effects on controlling the progression of DN [3]. Therefore, novel and effective therapeutic strategies are urgently needed for DN.

Renal tubular injury could cause interstitial fibrosis and tubulointerstitial proteinuria through inflammatory factors, which are typical features of DN [4, 5]. Ferroptosis is a type of cell death that is primarily induced by an excess of ferrous ions. This excess drives the production of lipid peroxidation via the Fenton reaction, ultimately triggering cell death [6]. Dixon et al. demonstrated through electron microscopy that erastin-induced ferroptosis in cells led to distinctive morphological changes including mitochondrial crinkling and increased membrane density. These alterations can separate ferroptosis from apoptosis, necrosis, and autophagy [7]. Feng et al. discovered that intervention with ferrostatin-1, a specific small-molecule inhibitor of ferroptosis, reduced the expression of kidney injury molecule-1 (KIM-1) and neutrophil gelatinase-associated lipocalin (NGAL) in renal tissues and urine of db/db mice. This finding demonstrates that inhibiting ferroptosis attenuates renal tubular injury [8].

Solute carrier family 7, member 11(SLC7A11) is one of the components of the system Xc, which transports cystine into the cell for the synthesis of glutathione (GSH). GSH is utilized by the enzyme glutathione peroxidase 4 (GPX4) to reduce lipid hydroperoxide within the biological membrane and convert it into a nontoxic lipid alcohol. This process can prevent the formation of lipid-reactive oxygen species (L-ROS) [9–11]. The SLC7A11/GSH/GPX4 axis is considered the classical signaling pathway that regulates ferroptosis. A significant reduction of expression of SLC7A11 and GPX4 was observed in kidney biopsy samples from diabetic patients [12, 13]. In streptozotocin-induced DN rats and high glucose-cultured tubular epithelial cells (TECs), the expressions of GPX4 and SLC7A11, and GSH activity, were reduced, while the expressions of malondialdehyde (MDA), KIM-1, and NGAL were elevated [14, 15]. Thus, the inhibition of ferroptosis, regulated by the SLC7A11/GSH/GPX4 axis, plays a crucial role in renal tubular injury in DN.

Tangshenning (TSN) is an effective formula for treating DN. It has proved safe and effective for DN treatment by the multicenter, randomized, double-blind clinical trial [16]. TSN was developed based on the principles of the collateral disease theory in traditional Chinese medicine (TCM). TSN is so beneficial to the Qi, tonifying the kidneys, dispelling stasis, dredging the collaterals, and descending the turbidity. TSN mainly consisted of *Astragalus mongholicus* Bunge (Chinese name: Huangqi), *Rheum palmatum* L. (Chinese name: Dahuang), *Ligusticum chuanxiong* Hort. (Chinese name: Chuanxiong), and *Rosa laevigata* Michx. (Chinese name: Jinyingzi). The pharmacological evidence has confirmed that several active ingredients in TSN, including quercetin, kaempferol, ferulic acid, astragaloside IV, and rhein, exhibit anti-epithelial-mesenchymal transition (EMT), anti-renal fibrosis, anti-oxidative stress, and anti-inflammatory effects in animal models of DN [17–21]. Our previous study demonstrated that TSN improved renal fibrosis and podocyte injury in animal models of DN [22, 23]. However, the therapeutic effects and mechanisms of TSN on renal tubular injury in DN remain unclear. In this study, we investigated whether TSN attenuates renal tubular injury by inhibiting ferroptosis regulated by the SLC7A11/GSH/GPX4 axis. This study provides a new perspective for understanding the pathogenesis of DN and identifying the new targets for the pharmacological treatment of DN.

## Materials and methods

### Ultrahigh pressure liquid chromatography-quadrupole-time of flight mass spectrometry (UPLC-QTOF/MS) analysis of the TSN formula

The TSN was purchased by the Chinese Medicine Factory (Beijing Tcmages Pharmaceutical Co. Ltd., Beijing, China) and composed of Astragalus mongholicus Bunge (Chinese name: Huangqi), Rheum palmatum L. (Chinese name: Dahuang), Ligusticum chuanxiong Hort. (Chinese name: Chuanxiong), and Rosa laevigata Michx. (Chinese name: Jinyingzi). The chemical composition and serum pharmacochemistry of TSN were identified by UPLC-QTOF/MS. UPLC was conducted on a UPLC HSS T3 column (100 × 2.1 mm, 1.8 µm) at 35 ℃ and the injection volume was set at 10 μL, with gradient elution. The mass spectrometry was detected in the 50–1500 m/z range with MS^e^ as the scan mode. In the positive ESI mode, the capillary voltage was set to 2.0 kV and the cone hole voltage to 20 V. The source and desolvation temperatures were set to 120 and 500 °C, respectively. The cone hole and desolvation gas flow rates were set to 50 and 800 L/h, respectively. In the negative ESI mode, the capillary voltage was set to 2.5 kV and the cone hole voltage to 20 V. The source and desolvation temperatures were set to 125 and 400 °C, respectively. The cone hole and desolvation gas flow rates were set to 50 and 800 L/h, respectively.

### Laboratory animal models

This study protocol was approved by the Institutional Animal Care and Use Committee of the Capital Medical University (Ethics Number: AEEI-2017-039), which complies with the National Institutes of Health Guidelines for the Care and Use of Laboratory Animals. Eight-week-old male KK-Ay mice and male C57BL/6 J mice (SPF grade, from Beijing Huafukang Bioscience Co., Ltd., Beijing, China) were housed at constant room temperature (24 ± 1 °C) and humidity (60–70%). They were freely fed and watered under a standard 12-h light/dark cycle. KK-Ay mice were fed high-fat chow for 4 weeks to induce DN, while C57BL/6 J mice were fed standard chow as the normal control group (NC group, *n* = 8). After 4 weeks, the random blood glucose (RBG) of KK-Ay mice was higher than 16.7 mM, and the 24-h urinary microalbumin excretion rate (24-h UAER) was significantly higher than the NC group (*p* < 0.05), representing the successful construction of the DN model. Then, KK-Ay mice were randomly divided into four groups (*n* = 8): (1) The diabetic nephropathy control group (DN group) received distilled water via oral gavage for 10 weeks; (2) The TSN low dose group (LT group) received TSN (10 g/kg·d) via oral gavage for 10 weeks; (3) The TSN high dose group (HT group) received TSN (20 g/kg·d) via oral gavage for 10 weeks; (4) The valsartan treatment group (VAL group) received valsartan suspension solution (10 mg/kg·d) via oral gavage for 10 weeks. According to the standard body mass and body surface area conversion algorithm, the clinically equivalent dose of TSN for the mice was 10 g/kg·d. The detailed formula is as follows, (X mg/kg·d * 70 kg * 0.0026)/20 g = 9.1 * X mg/kg·d, where X mg/kg·d is the dose of human [24]. The high dose (20 g/kg·d) was twice the low dose (10 g/kg·d). RBG and microalbuminuria were dynamically monitored during the experiment. After a 10-week administration, serum was collected to determine renal function, and renal tissue was also collected for western blot, pathological staining, etc.

Serum pharmacology was conducted in vitro [25], and 8-week-old SD rats (purchased from Beijing Vital River Laboratory Animal Technology Co., Ltd., Beijing, China) were used for TSN-containing serum preparation. The TSN group was gavaged TSN (20 g/kg·d), while the normal control group received an equal amount of distilled water for 7 days. TSN-containing and blank serum were collected 1 h after the last administration, incubated in a water bath at 56 °C for 30 min, and stored at − 80 °C for subsequent in vitro experiments.

### Cell culture and experimental protocol

HK-2 cells (GDC0152) were purchased from the China Type Culture Collection (Wuhan, China) and cultured in DMEM low-glucose medium (Gibco, USA) containing 10% fetal bovine serum (Analysis Quiz, China) in an incubator at 37 °C and 5% CO_2_. HK-2 cells were divided into the following groups: (1) Normal group (NG): 5.5 mM glucose + blank serum. (2) Mannitol group (MG): 5.5 mM glucose + 24.5 mM D-mannitol + blank serum. (3) High glucose group (HG): 30 mM glucose + blank serum. (4) TSN treatment group (TSN): 30 mM glucose + 10% TSN-containing serum. (5) Ferrostatin-1 treatment group (Fer-1): 30 mM glucose + blank serum + 2 μM ferrostatin-1. (6) TSN + Erastin group (T + Era): 30 mM glucose + 10% TSN-containing serum + 2 μM erastin. Following cell attachment, the above intervention was performed for 48 h.

### Pathological examination

Fresh kidney tissues were fixed in 4% paraformaldehyde and then embedded in paraffin. Four μm thick tissue sections were transferred to glass slides for HE, PAS, and Prussian blue staining. Results were observed with an optical microscope (Nikon Eclipse Ti-U, Tokyo, Japan). Kidney tissue sections were also fixed in 2% glutaraldehyde for 2 h, and ultrathin sections were collected. Kidney ultrastructure was observed using a transmission electron microscope (TEM, HT7700, HITACHI, Japan).

### Western blot assay

Western blot was used to assess the protein levels of KIM-1, NGAL, SLC7A11, and GPX4. First, the total protein of kidney tissue and HK-2 cells were extracted, separated by polyacrylamide gel electrophoresis, and then transferred to the PVDF membrane. After blocking with 5% skim milk for 2 h at 37 °C, anti-KIM-1 (1:1000, ab47635, Abcam), anti-NGAL (1:1000, 26991-1-AP, Proteintech), anti-SLC7A11 (1:1000, ab37185, Abcam), and anti-GPX4 (1: 1000, 67763-1-Ig, Proteintech) were incubated on the membrane overnight. Then, the membrane was incubated with HRP-conjugated secondary antibody for 1 h at 37 °C. Finally, membranes were exposed using a gel imaging system (FUSION FX6 XT, Vilber, France), and gray value analysis was performed using the ImageJ software.

### Immunofluorescence

HK-2 cells were seeded into a 24-well plate, followed by different administration for 48 h. After washing three times with PBS, they were fixed in 4% paraformaldehyde solution for 15 min. Cells were blocked with 10% goat serum for 45 min after incubation with 0.05% Triton X-100 for 10 min. Then, cells were incubated with anti-KIM-1 (1:200, ab47635, Abcam) and anti-SLC7A11 (1:200, ab37185, Abcam) overnight at 4 °C. On the next day, after washing with PBS, cells were incubated with fluorescent secondary antibodies for 1 h at 37 °C in the dark. Subsequently, nuclei were stained with DAPI. Finally, images were observed under a fluorescence microscope (Nikon ECLIPSE Ti-U, Tokyo, Japan). Three fields were randomly selected from each slide and statistical analysis was performed with Image-Pro Plus software.

### Cellular ROS analysis

Intracellular ROS levels were evaluated using the DCFH-DA probe (S0033, Beyotime, Shanghai, China), and the fluorescence intensity indirectly reflects the degree of oxidative stress in cells. HK-2 cells were seeded in 6-well plates and given different treatments for 48 h. After adding the DCFH-DA probe (10 μM) to cells, they were incubated for 25 min at 37 °C in the dark. Afterward, images were observed and collected under a fluorescence microscope (Nikon ECLIPSE Ti-U, Tokyo, Japan). Finally, we randomly selected three fields of view for fluorescence intensity analysis by Image-Pro Plus software.

### Iron assay

The level of ferrous ions in kidney tissues or HK-2 cells was measured using the Iron Assay Kit (BC5415, Solarbio, Beijing, China) or (K390-100, BioVision, USA), respectively. Samples were prepared according to the manufacturer’s instructions. Finally, a microplate reader (SpectraMax iD3, SpectraMax, United States) was used to measure the absorbance at 593 nm.

### GSH and MDA assays

The GSH content and GSH/Oxidized Glutathione (GSSG) ratio in kidney tissues or HK-2 cells were measured with GSH/GSSG Assay Kit (BC1175/BC1185, Solarbio, Beijing, China) or (A061-1, Nanjing Jiancheng Institute of Biological Engineering, Nanjing, China), respectively, following the manufacturer’s instructions. The GSH/GSSG ratio was calculated as GSH/GSSG.

The MDA content in kidney tissues or HK-2 cells was measured using the MDA assay kit (BC0025, Solarbio, Beijing, China) or (A003-1, Nanjing Jiancheng Institute of Biological Engineering, Nanjing, China), respectively. Briefly, samples were mixed with the reagents according to the instructions. The absorbance of the supernatant was measured at 532 nm using a microplate reader (SpectraMax iD3, SpectraMax, United States).

### Cystine assay

The levels of cystine in HK-2 cells were measured using the Cystine Uptake Fluorometric Assay Kit (E-BC-F066, Elabscience, China). The intervention samples in each group were divided into measurement and control tubes. Afterward, the samples were processed and incubated according to instructions. A fluorescence microplate reader was utilized to measure the fluorescence values of each group of supernatants, using an excitation wavelength of 485 nm and an emission wavelength of 535 nm.

### Cell viability

The cell viability was assayed by the Cell Counting Kit-8 kit (CCK-8) (C0037, Beyotime, Shanghai, China). After inoculating HK-2 cells in 96-well plates, different interventions were given for 48 h. Each well was incubated with 10 μL of CCK-8 solution at 37 °C for 2 h. Finally, the optical density (OD) at 450 nm was measured by a microplate reader (SpectraMax iD3, SpectraMax, United States).

### Statistical analysis

SPSS 19.0 software (IBM Corporation, Armonk, NY, USA) was used for statistical analysis. Data are presented as means ± standard deviations (SD). One-way ANOVA was used for comparisons between multiple groups. A *p* < 0.05 was considered statistically significant.

## Results

### Chemical composition and serum pharmacochemical analysis of TSN

**Fig. 1:**
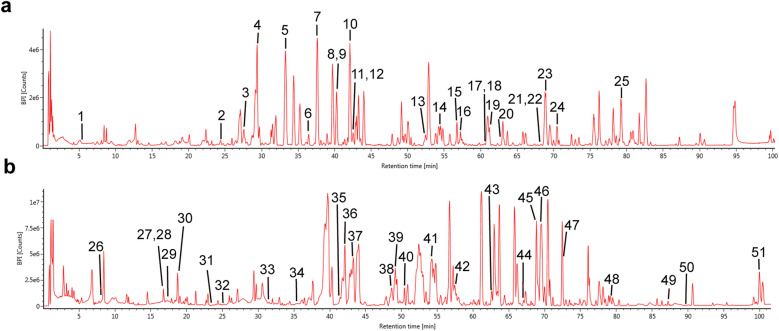
Chemical composition of Tangshenning (TSN). **a** Base peak ion (BPI) chromatogram of the TSN formula in the positive mode. **b** BPI chromatogram of the TSN formula in the negative mode. Labeled numbers correspond to Table S1

**Fig. 2:**
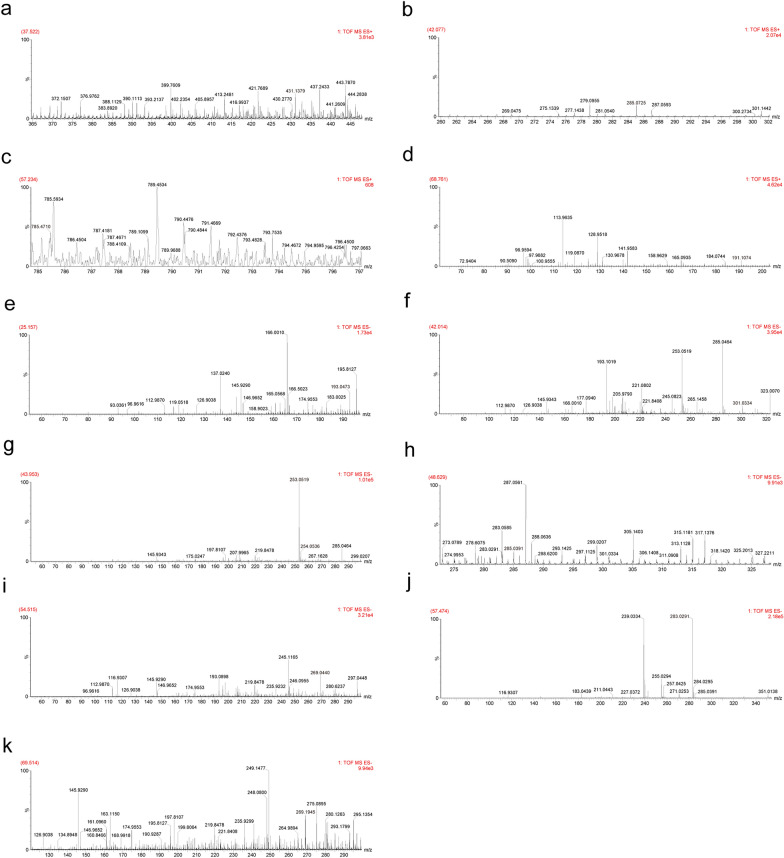
Chemical composition of Tangshenning (TSN)-containing serum. Mass spectra of **a** ononin, **b** calycosin, **c** astragaloside IV, **d** ligustilide, **e** ferulic acid, **f** quercetin, **g** chrysophanol, **h** kaempferol, **i** aloeemodin, **j** rhein, and **k** emodin

First, we evaluated the chemical composition and serum medicinal chemistry of TSN using UPLC-QTOF/MS. After analysis, 51 compounds were identified in TSN (Table S1), and 11 components were identified in TSN-containing serum (Table 1). The base peak ion (BPI) chromatograms of TSN in both positive and negative ion (ESI) modes are shown in Fig. 1. The mass spectra of the 11 components identified in the serum containing TSN are presented in Fig. 2. The BPI chromatograms of TSN-containing and blank serum are depicted in Figure S1.

**Table 1:** Chemical composition of Tangshenning (TSN)-containing serum

No	Observed RT (min)	Component name	Formula	Neutral mass (Da)	Mass error (ppm)	ESI mode
1	37.522	Ononin	C_22_H_22_O_9_	430.12638	3.8	Positive
2	42.077	Calycosin	C_16_H_12_O_5_	284.06847	12	Positive
3	57.234	Astragaloside IV	C_41_H_68_O_14_	784.46091	2.9	Positive
4	68.761	Ligustilide	C_12_H_14_O_2_	190.09938	6.3	Positive
5	25.157	Ferulic acid	C_10_H_10_O_4_	194.05791	5.1	Negative
6	42.014	Quercetin	C_15_H_10_O_7_	302.04265	5.1	Negative
7	43.953	Chrysophanol	C_15_H_10_O_4_	254.05791	5	Negative
8	48.629	Kaempferol	C_15_H_10_O_6_	286.04774	1.7	Negative
9	54.515	Aloeemodin	C_15_H_10_O_5_	270.05282	4.3	Negative
10	57.474	Rhein	C_15_H_8_O_6_	284.03209	4.2	Negative
11	69.514	Emodin	C_15_H_10_O_5_	270.05282	4.3	Negative

### Effects of TSN on 24-h UAER, RBG, renal function, and relative kidney weight in DN mice

**Fig. 3:**
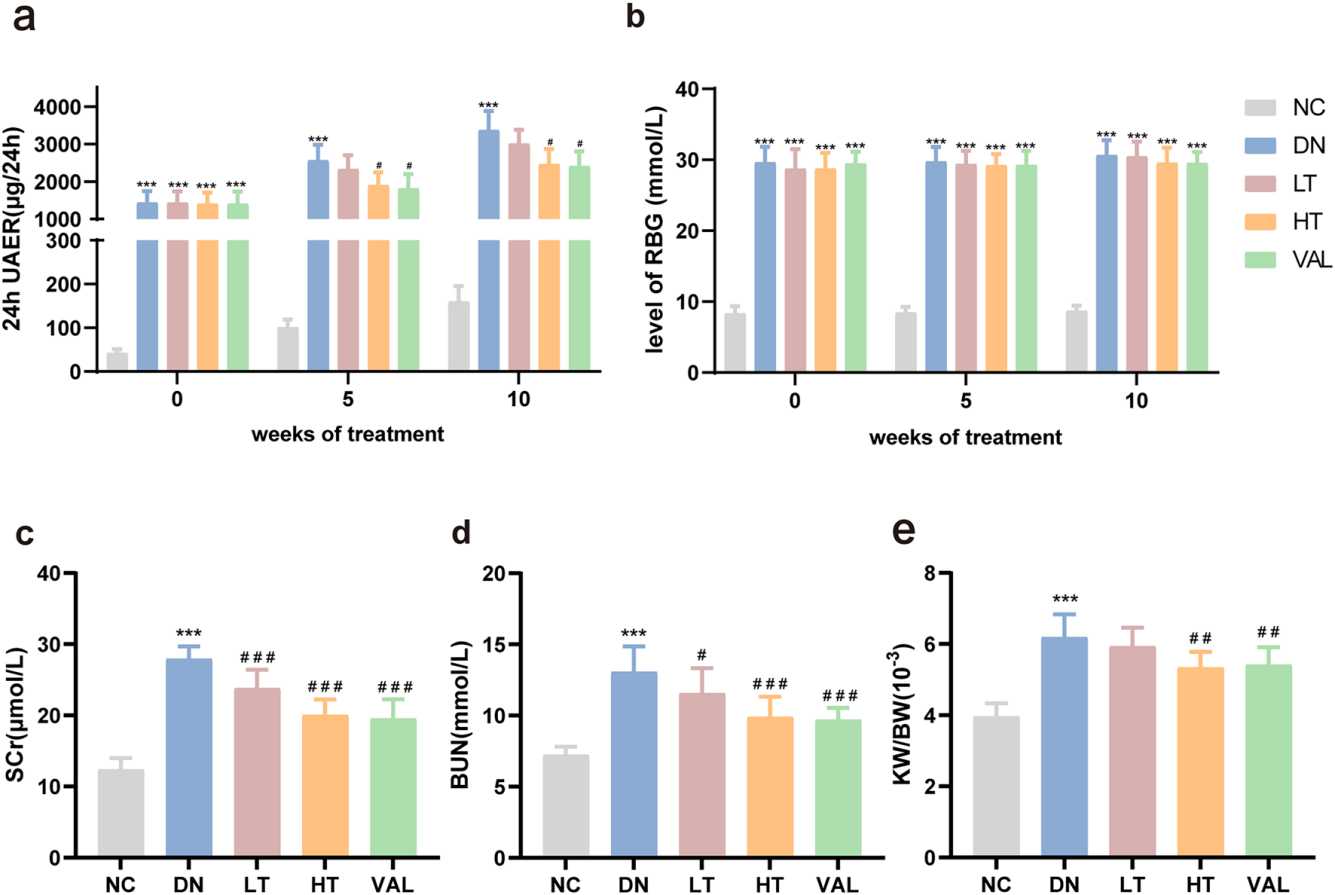
Effects of TSN on 24-h UAER, RBG, renal function, and relative kidney weight in DN mice. **a** 24-h UAER. **b** RBG. **c** Serum creatinine (SCr). **d** Blood urea nitrogen (BUN). **e** Relative kidney weight (kidney weight/body weight). NC: normal control group. DN: diabetic nephropathy control group. LT: TSN low dose group. HT: TSN high dose group. VAL: Valsartan treatment group. Data are shown as means ± standard deviations (*n* = 8). *** *p* < 0.001, compared with the NC group; ^#^ *p* < 0.05, ^##^ *p* < 0.01, ^###^ *p* < 0.001, compared with the DN group

To evaluate the effect of TSN on DN, KK-Ay mice were intragastrically administered low dose (10 g/kg·d) and high dose (20 g/kg·d) of TSN in vivo, along with valsartan as a positive control. We found that the high-dose TSN significantly reduced the 24-h urinary albumin excretion rate (UAER) at 5 and 10 weeks of administration (Fig. 3a), but had no significant effect on random blood glucose (RBG) (Fig. 3b). Additionally, the serum creatinine (SCr) and blood urea nitrogen (BUN) levels, and the relative kidney weight of KK-Ay mice, were dramatically reduced after 10 weeks of high-dose TSN treatment (Fig. 3c–e). These results suggested that TSN may reduce proteinuria, protect renal function, and inhibit renal hypertrophy in DN. Furthermore, the effect was better in the high-dose TSN group (HT).

### Effects of TSN on renal pathology and tubular injury in DN mice

**Fig. 4:**
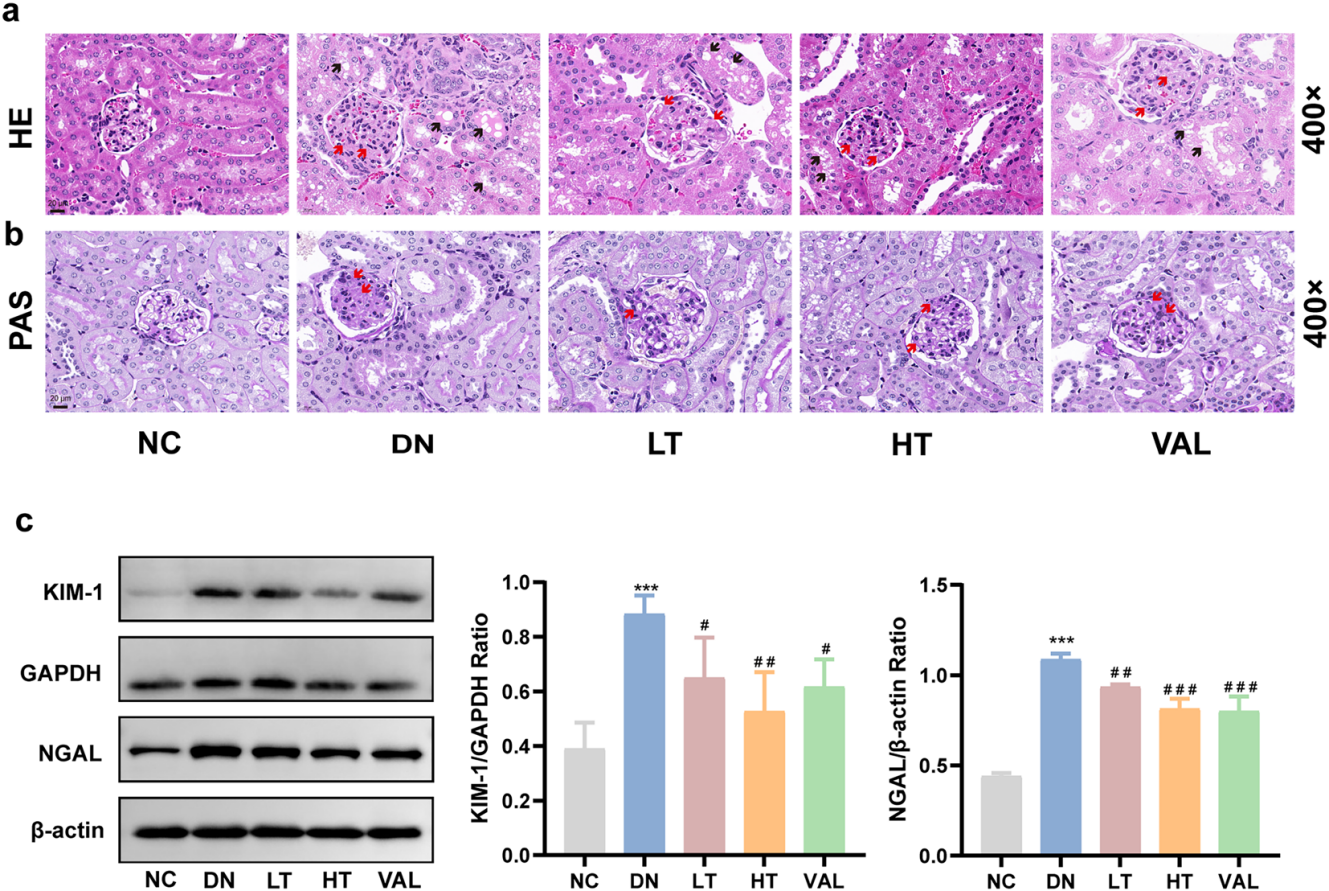
Effects of TSN on renal pathology and tubular injury in DN mice. **a** HE staining of renal tissue (×400, Scale bar: 20 μm). Red arrows indicate mesangial cell proliferation, mesangial matrix deposition. Black arrows indicate protein casts, vacuolar degeneration, proximal tubule microvilli shedding. **b** PAS staining of renal tissue (×400, Scale bar: 20 μm). Red arrows indicate glycogen deposition. **c** Western blot bands and quantitative analysis of KIM-1 and NGAL protein levels in renal tissue. NC: normal control group. DN: diabetic nephropathy control group. LT: TSN low dose group. HT: TSN high dose group. VAL: Valsartan treatment group. Data are shown as means ± standard deviations (*n* = 3). *** *p* < 0.001, compared with the NC group; ^#^ *p* < 0.05, ^##^ *p* < 0.01, ^###^ *p* < 0.001, compared with the DN group

HE and PAS staining were used to observe renal tissue structure and assess the level of glycogen deposition. HE staining showed that mice in the DN group presented typical pathological lesions, including mesangial cell proliferation, mesangial matrix deposition, microvillous shedding of the proximal tubules, tubular vacuolar degeneration, and protein casts (Fig. 4a). PAS staining showed a large amount of glycogen protein deposition in the kidneys of DN mice (Fig. 4b). However, these changes were significantly alleviated in the kidneys of mice that received TSN (Fig. 4a, b). Meanwhile, to investigate the protective effect of TSN on tubular function, western blot were used to analyze the expression of two sensitive proteins of tubular injury in renal tissue. The results showed that treatment with TSN significantly reversed the elevated KIM-1 and NGAL levels in the DN group, with TSN at high dose having the best effect (Fig. 4c). It was concluded that TSN could significantly reduce tubular injury and renal pathological injury in DN mice.

### TSN inhibits ferroptosis and activates SLC7A11/GSH/GPX4 axis in the renal tissues of DN mice

**Fig. 5:**
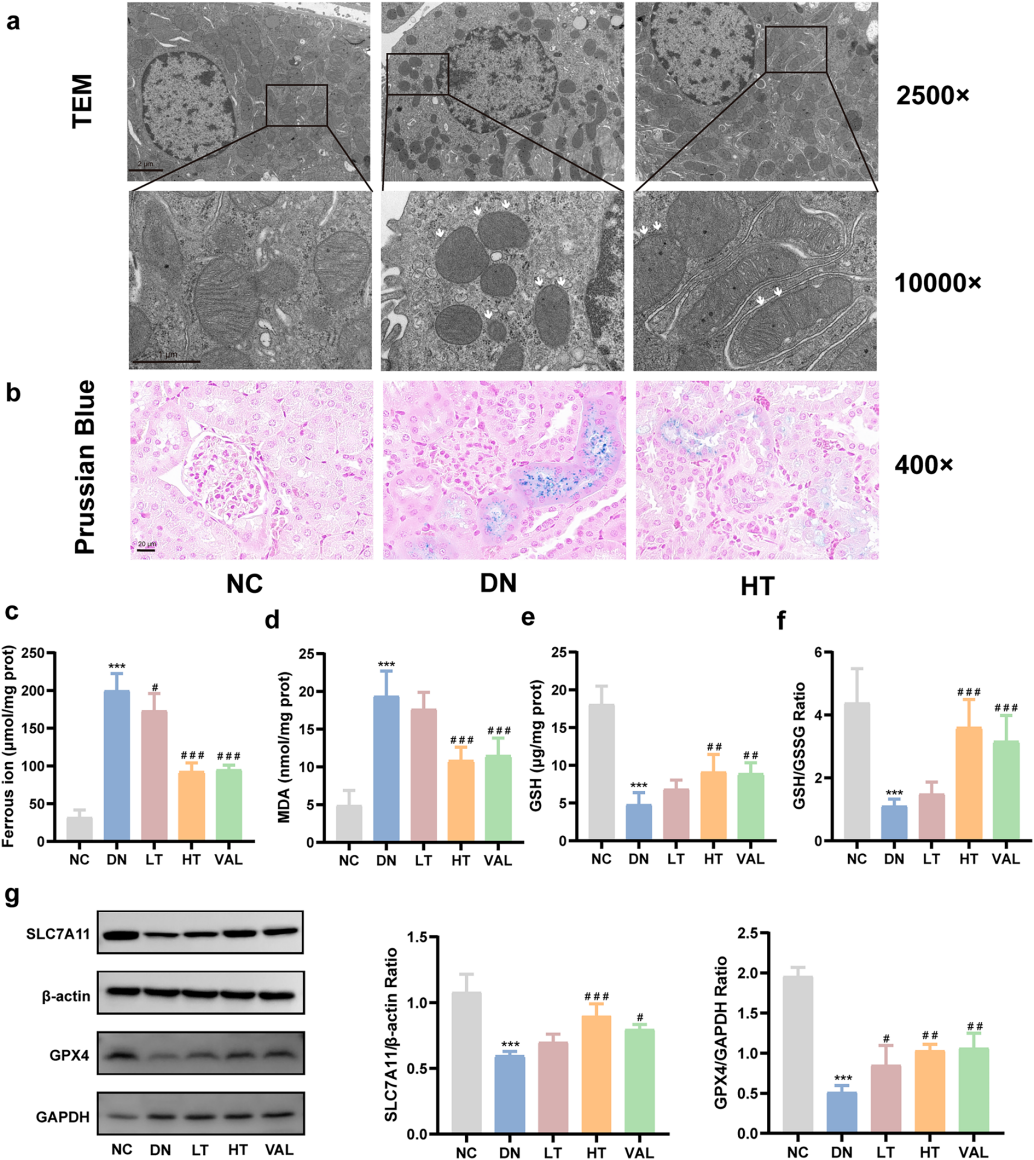
Effects of TSN on ferroptosis and the SLC7A11/GSH/GPX4 axis in renal tissue of DN mice. **a** Electron microscope observation of ferroptosis in renal tissue (×2500, Scale bar: 2 μm, ×10,000, Scale bar: 1 μm). White arrows indicate increased mitochondrial membrane density, reduced or absent mitochondrial cristae, and shrinkage of mitochondria. **b** Prussian blue staining (×400, Scale bar: 20 μm). **c** Ferrous ion content (*n* = 5). **d** MDA content (*n* = 5). **e** GSH content (*n* = 5). **f** GSH/GSSG ratio (*n* = 5). **g** Western blot bands and quantitative analysis of SLC7A11 and GPX4 protein levels (*n* = 3). NC: normal control group. DN: diabetic nephropathy control group. LT: TSN low dose group. HT: TSN high dose group. VAL: Valsartan treatment group. Data are shown as means ± standard deviations. *** *p* < 0.001, compared with the NC group; ^#^ *p* < 0.05, ^##^ *p* < 0.01, ^###^ *p* < 0.001, compared with the DN group

To validate TSN’s suppression of ferroptosis, we monitored the mitochondria status in the tubular region of the kidney tissue. Transmission electron microscopy showed that renal tubules from DN mice possessed typical morphological features of ferroptosis, such as shrunken mitochondrial size, increased membrane density, and reduced or absent mitochondrial cristae (Fig. 5a). The Prussian Blue staining revealed that iron was mainly concentrated in the renal tubules, indicating that the renal tubules might be a crucial site of ferroptosis (Fig. 5b). Interestingly, the ferroptosis-related characteristics were significantly reduced in the TSN group (Fig. 5a, b). The next results showed that the levels of ferrous ion and MDA, markers of ferroptosis, were significantly increased, and both GSH levels and the ratio of GSH to GSSG were distinctly decreased in the DN group, compared with the NC group. Changes in these parameters were reversed after TSN treatment (Fig. 5c–f). In addition, the levels of ferroptosis inhibitory proteins SLC7A11 and GPX4 were notably lower in the DN group compared with the NC group, but were conspicuously increased after TSN treatment (Fig. 5g). Accordingly, we reasonably speculated that TSN reduced DN tubular injury may be related to the inhibition of ferroptosis.

### Ferroptosis inhibition protects HK-2 cells injury induced by high glucose

**Fig. 6:**
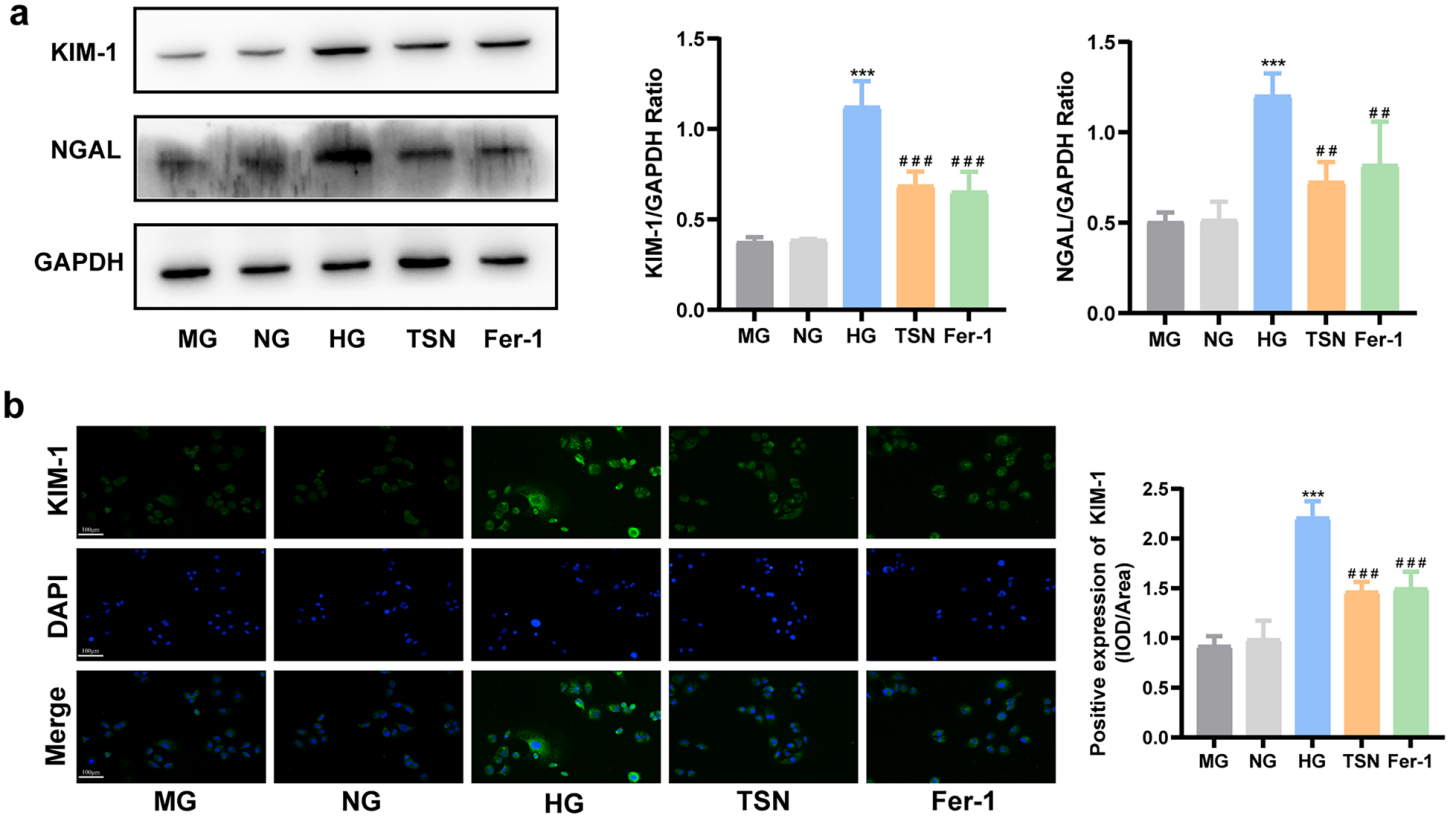
Effects of TSN on high glucose-induced HK-2 cells injury. **a** Western blot bands and quantitative analysis of KIM-1 and NGAL protein levels. **b** Immunofluorescence images and quantitative analysis of KIM-1 protein levels (×200, Scale bar: 100 μm). MG: mannitol group. NG: normal group. HG: high glucose group. TSN: TSN treatment group. Fer-1: ferrostatin-1 treatment group. Data are shown as means ± standard deviations (*n* = 3). *** *p* < 0.001, compared with the NG group; ^#^ *p* < 0.05, ^##^ *p* < 0.01, ^###^ *p* < 0.001, compared with the HG group

**Fig. 7:**
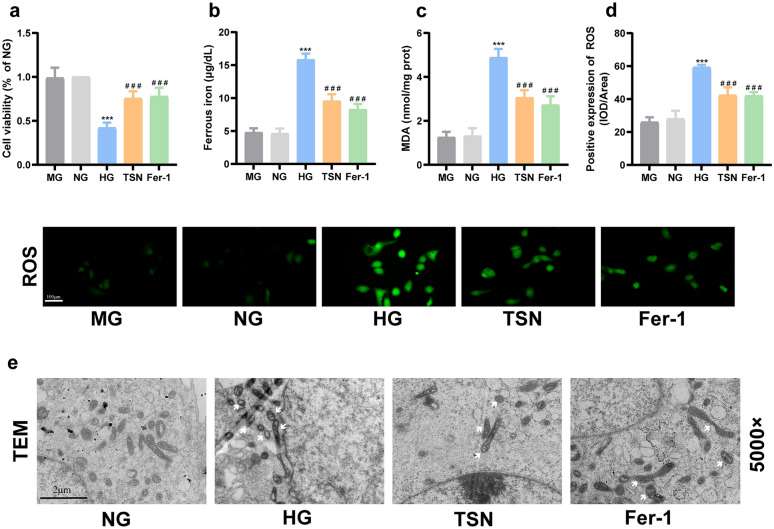
Effects of TSN on high glucose-induced HK-2 cells ferroptosis. **a** Cell viability. **b** Ferrous ion content. **c** MDA content. **d** Representative images and quantitative analysis of ROS (×200, Scale bar: 100 μm). **e** Transmission electron microscope (TEM) observation of ferroptosis in HK-2 cells (×5000, Scale bar: 2 μm). White arrows indicate increased membrane density of mitochondria and reduced or absent mitochondrial cristae. MG: mannitol group. NG: normal group. HG: high glucose group. TSN: TSN treatment group. Fer-1: ferrostatin-1 treatment group. Data are shown as means ± standard deviations (*n* = 3). *** *p* < 0.001, compared with the NG group; ^#^ *p* < 0.05, ^##^ *p* < 0.01, ^###^ *p* < 0.001, compared with the HG group

We had shown that TSN inhibited ferroptosis in DN mice. Next, we investigated the impact of ferroptosis on renal tubular injury in DN using HK-2 cell lines cultured in high glucose conditions. The protein levels of KIM-1 and NGAL significantly increased in HK-2 cells incubated with high glucose, indicating the successful establishment of the renal tubular injury model. Similar to the ferrostatin-1 intervention, the levels of KIM-1 and NGAL proteins were significantly reduced after the TSN treatment, indicating that the inhibition of ferroptosis mitigates renal tubular injury (Fig. 6a, b). In addition, the intervention with mannitol did not affect the levels of KIM-1 and NGAL (*p* > 0.05), indicating that the observed effect of high glucose treatment on HK-2 cells was independent of any increase in the osmotic pressure. The diminished cell viability in high glucose conditions was restored by TSN treatment, in line with the protective impact of ferrostatin-1 (Fig. 7a). Additionally, TSN decreased ferrous ion and MDA production in HK-2 cells cultured with high glucose. (Fig. 7b, c). Consistently, DCFH-DA staining showed that TSN treatment reduced ROS levels in HK-2 cells stimulated by high glucose (Fig. 7d). High glucose-induced HK-2 cells also exhibited ultrastructural damage, such as mitochondrial crinkling and increased membrane density, which were mitigated by TSN or ferrostatin-1 treatment (Fig. 7e). Taken together, the nephroprotection of TSN, similar to the inhibitor of ferroptosis, can attenuate the injury to HK-2 cells induced by high glucose.

### TSN activates SLC7A11/GSH/GPX4 axis in HK-2 cells incubated with high glucose

**Fig. 8:**
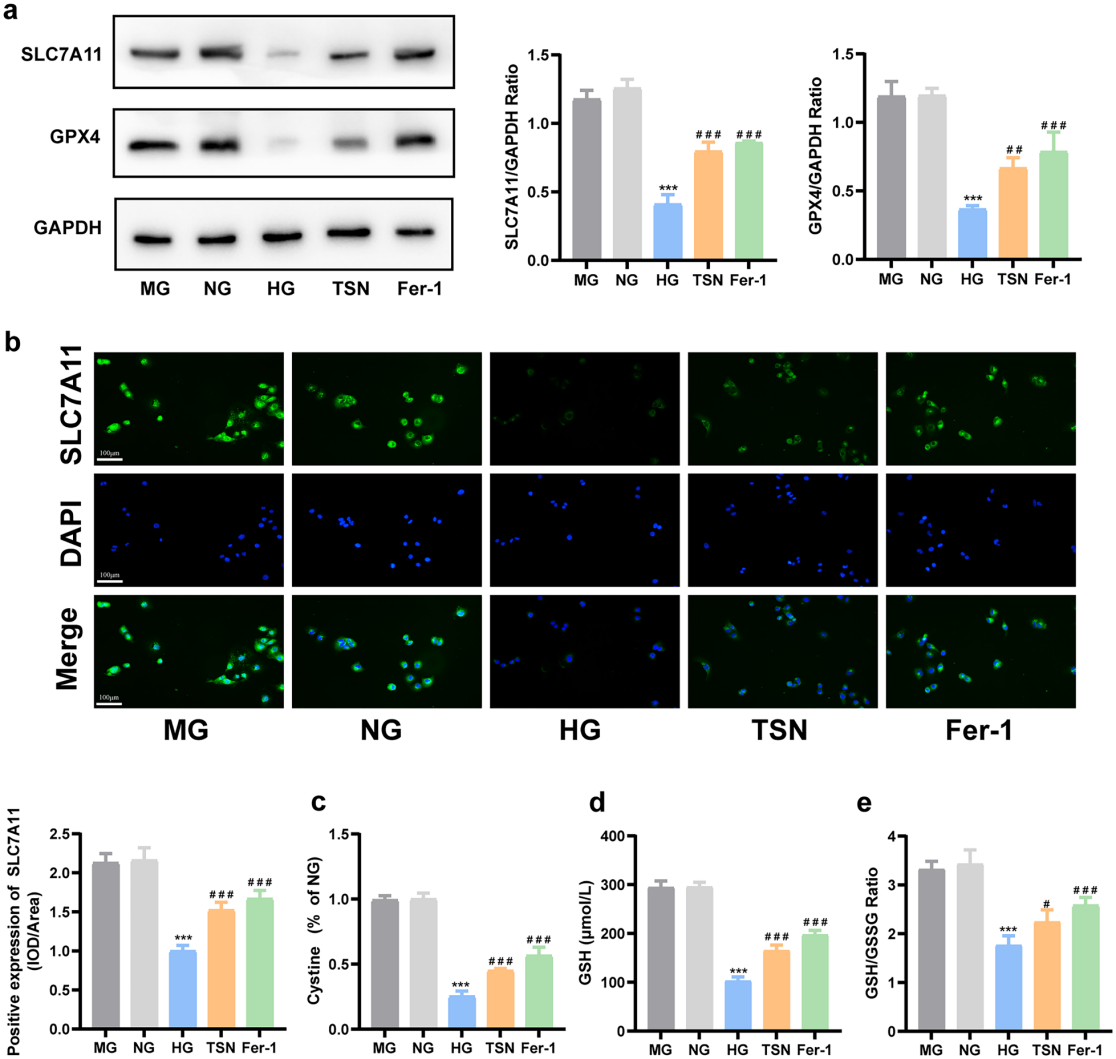
Effects of TSN on SLC7A11/GSH/GPX4 axis in high glucose-induced HK-2 cells injury model. **a** Western blot bands and quantitative analysis of SLC7A11 and GPX4 protein levels. **b** Immunofluorescence images and quantitative analysis of SLC7A11 protein levels (×200, Scale bar: 100 μm). **c** Fluorescence intensity of cystine. **d** GSH content. **e** GSH/GSSG ratio. MG: mannitol group. NG: normal group. HG: high glucose group. TSN: TSN treatment group. Fer-1: ferrostatin-1 treatment group. Data are shown as means ± standard deviations (*n* = 3). *** *p* < 0.001, compared with the NG group; ^#^ *p* < 0.05, ^##^ *p* < 0.01, ^###^ *p* < 0.001, compared with the HG group

Consistent with the results of the DN mice model, the western blot showed that the protein expression of SLC7A11 and GPX4 strikingly reduced in the HG group, and TSN reversed this change (Fig. 8a). The results of immunofluorescence confirmed the localization and alterations of SLC7A11 as well (Fig. 8b). Furthermore, TSN reversed the decrease in the intake of cystine, GSH content, and GSH/GSSG ratio under high glucose conditions, achieving similar effects to those of ferrostatin-1 (Fig. 8c–e). The above experiments further confirmed that TSN can activate the SLC7A11/GSH/GPX4 axis in HK-2 cells incubated with high glucose.

### TSN protected HK-2 cells from ferroptosis induced by high glucose via the SLC7A11/GSH/GPX4 axis

**Fig. 9:**
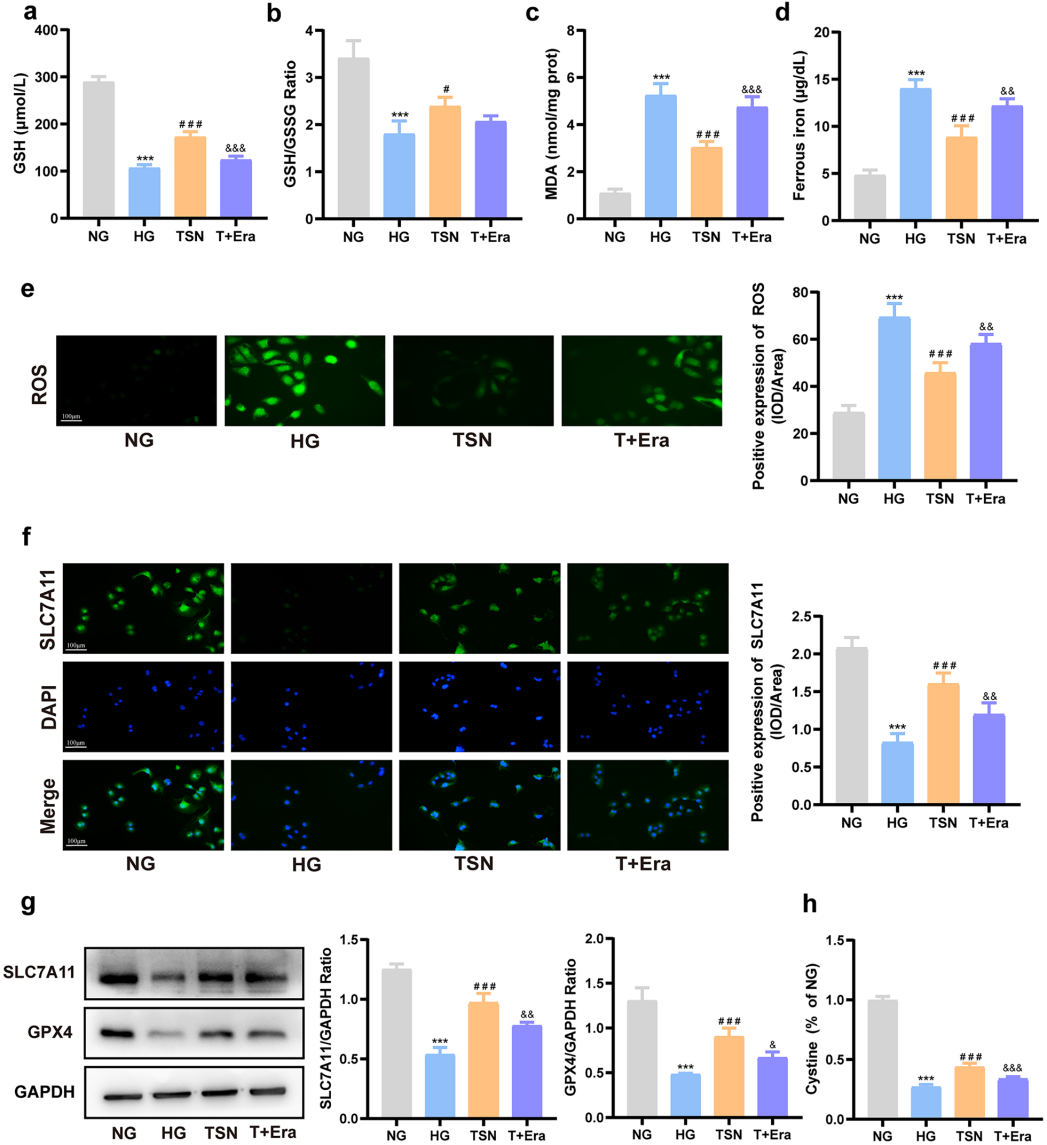
TSN protected HK-2 cells from ferroptosis induced by high glucose by regulating the SLC7A11/GSH/GPX4 axis. **a** GSH content. **b** GSH/GSSG ratio. **c** MDA content. **d** Ferrous ion content. **e** Representative images and quantitative analysis of ROS (×200, Scale bar: 100 μm). **f** Immunofluorescence images and quantitative analysis of SLC7A11 protein levels (×200, Scale bar: 100 μm). **g** Western blot bands and quantitative analysis of SLC7A11 and GPX4 protein levels. **h** Fluorescence intensity of cystine. NG: normal group. HG: high glucose group. TSN: TSN treatment group. T + Era: TSN + Erastin group. Data are shown as means ± standard deviations (*n* = 3). *** *p* < 0.001, compared with the NG group; ^#^ *p* < 0.05, ^##^ *p* < 0.01, ^###^ *p* < 0.001, compared with the HG group; ^&^ *p* < 0.05, ^&&^ *p* < 0.01, ^&&&^ *p* < 0.001, compared with the TSN group

**Fig. 10:**
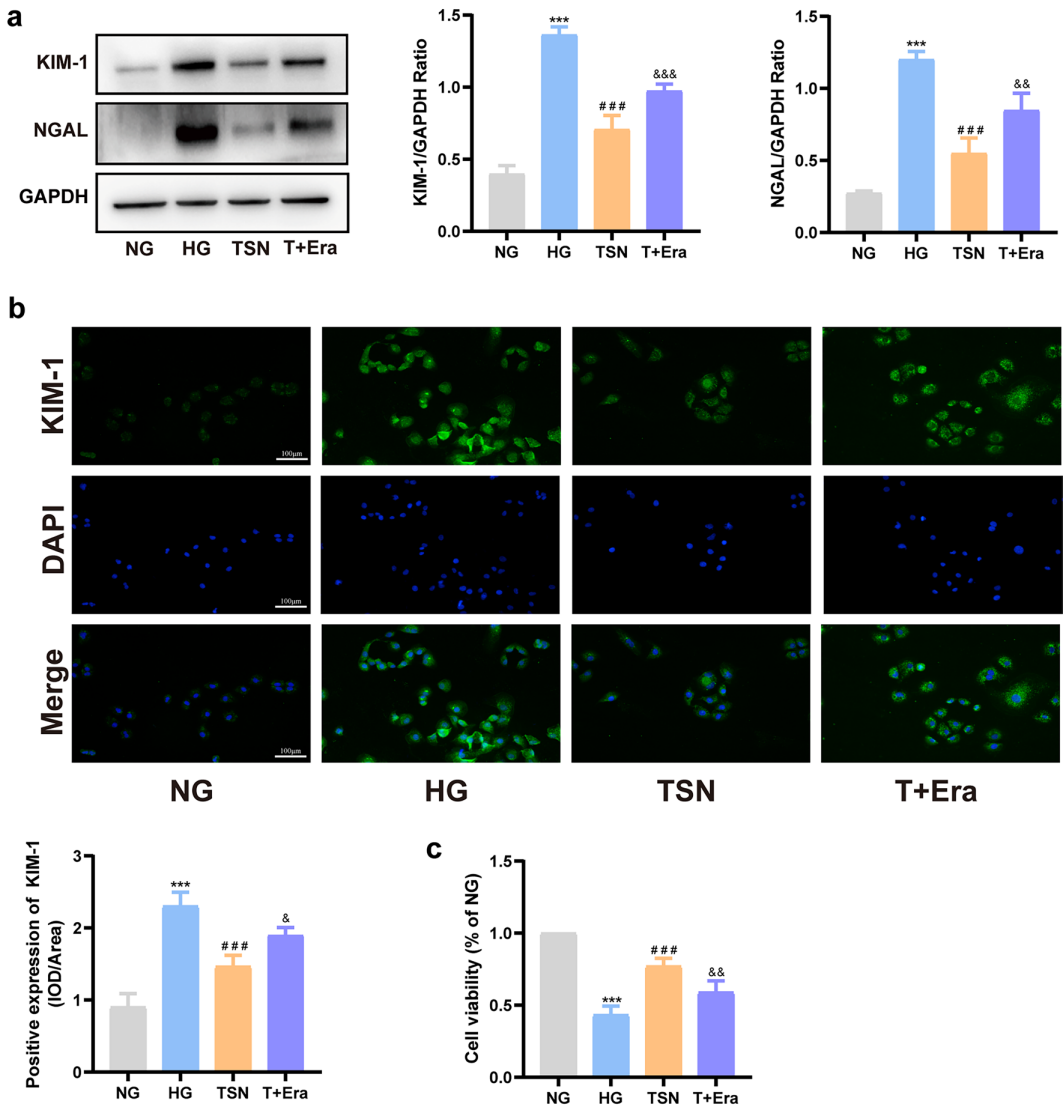
TSN protected HK-2 cells from injury induced by high glucose by inhibiting ferroptosis. **a** Western blot bands and quantitative analysis of KIM-1 and NGAL protein levels. **b** Immunofluorescence images and quantitative analysis of KIM-1 protein levels (×200, Scale bar: 100 μm). **c** Cell viability. NG: normal group. HG: high glucose group. TSN: TSN treatment group. T + Era: TSN + Erastin group. Data are shown as means ± standard deviations (*n* = 3). *** *p* < 0.001, compared with the NG group; ^#^ *p* < 0.05, ^##^ *p* < 0.01, ^###^ *p* < 0.001, compared with the HG group; ^&^ *p* < 0.05, ^&&^ *p* < 0.01, ^&&&^ *p* < 0.001, compared with the TSN group

To further illustrate how TSN inhibits ferroptosis and attenuates high glucose-induced damage in HK-2 cells, we utilized a ferroptosis inducer (Erastin). The combined intervention of erastin and TSN inhibited the effects of TSN on elevating intake of cystine, GSH content, SLC7A11 and GPX4 protein levels (Fig. 9a, f–h). Furthermore, erastin counteracted the effects of TSN in reducing levels of ROS, MDA, and ferrous iron (Fig. 9c–e). Prominently, the combined erastin and TSN intervention hindered the impact of TSN on reducing KIM-1 and NGAL protein levels (Fig. 10a, b). The ability of TSN to enhance cell viability, which decreases under high glucose conditions, was also inhibited by erastin (Fig. 10c). These results provide additional evidence that TSN mitigates the high glucose-induced renal tubular epithelial cell damage by inhibiting ferroptosis through the regulation of the SLC7A11/GSH/GPX4 axis.

## Discussion

TSN, consisting of Astragalus mongholicus Bunge (Chinese name: Huangqi), Rheum palmatum L. (Chinese name: Dahuang), Ligusticum chuanxiong Hort. (Chinese name: Chuanxiong), and Rosa laevigata Michx. (Chinese name: Jinyingzi), has been used clinically in DN for many years. In the current study, we utilized UPLC-QTOF/MS to characterize the active compounds in TSN powder and TSN-containing serum. We identified 51 components in TSN powder, of which 11 components including astragaloside IV, calycosin, emodin, ligustilide, rhein, and quercetin entered the serum to exert their effects. Previous studies showed that calycosin, astragaloside IV, emodin, and ligustilide were the main active components of TSN. Their contents were 104.9 μg/g, 62.9 μg/g, 40.7 μg/g, and 22.8 μg/g, respectively [26]. Astragaloside IV, ligustilide, and emodin, were shown to protect renal function and reduce proteinuria [20, 27, 28]. Calycosin, astragaloside IV, and rhein were evidenced in the attenuation of renal tubular injury in DN [18, 20, 29]. Surprisingly, quercetin, emodin, rhein, calycosin, and astragaloside IV were demonstrated to play a protective role in DN through ferroptosis [29–33]. These results provide a comprehensive material basis for studying the mechanism of TSN in the DN treatment. In addition, in future studies, we will perform further experiments in vivo and in vitro to verify the efficacy and therapeutic mechanism of major compounds.

Our previous study about TSN focused on its improvement of glomerular intrinsic cells [26], but its effects on DN tubular injury is unclear. However, based on the multi-component and multi-target characteristics of TSN, a comprehensive study of it is necessary. Therefore, this study was undertaken in an attempt to bridge this knowledge gap. To further confirm the role of TSN in treating DN renal tubular injury, we employed the KK-Ay mouse model for validation. It is recognized that KIM-1 and NGAL are both markers of tubular injury in DN [34, 35]. We have shown that TSN can downregulate KIM-1 and NGAL protein levels in renal tissue, indicating its potential to reduce renal tubular injury. The results of HE staining and PAS staining corroborated this conclusion. Interestingly, the high-dose effect of TSN was significantly better than the low-dose.

Ferroptosis is a type of cell death triggered by an excess of ferrous ions that induce lipid peroxidation reactions [9]. A previous clinical study on TSN has confirmed that its therapeutic efficacy is associated with improved oxidative stress in DN patients [16]. Meanwhile, previous network pharmacological studies have also shown that oxidative stress is a pivotal point in TSN improving DN [36]. To elucidate the molecular mechanism by which TSN attenuates DN tubular injury, we examined relevant indicators of ferroptosis in animal models. Here, the Prussian blue staining revealed that iron ions were mainly deposited in the renal tubular region in DN mice. The ultrastructure observed under electron microscopy revealed the typical morphological characteristics of ferroptosis in the renal tubular region. These findings were similar to the results obtained in the T2DM mouse model [37]. The results indicated that iron accumulation and cellular ferroptosis primarily occurred in DN renal tubules. Surprisingly, TSN treatment reversed these changes in the structure of renal tubular mitochondria. Furthermore, after TSN treatment, the increase of ferrous ion and MDA, and the decrease of GSH were reversed. In summary, TSN mitigated renal tubular injury in DN mice, similar to the effects of the ferroptosis inhibitor ferrostatin-1 [8, 38].

To further clarify the role of ferroptosis in DN renal tubular injury, we used ferrostatin-1 as a positive control agent in vitro. The levels of ferrous ion, ROS, and MDA in HK-2 cells incubated with high glucose decreased by ferrostatin-1 intervention, which is consistent with the study published by Li et al. [38]. Our experiments showed that TSN inhibits lipid peroxidation and cell ferroptosis induced by high glucose. Subsequently, we measured KIM-1 and NGAL protein levels using immunofluorescence and western blot analysis. The data showed that TSN attenuated high glucose-induced renal tubular epithelial cell injury, similar to the effects of ferrostatin-1. Huang et al. [29] also demonstrated that calycosin, the primary component of TSN, inhibits ferroptosis, protects renal function, and reduces tubular damage, providing evidence of the role of TSN in DN treatment.

The SLC7A11/GSH/GPX4 axis is a vital pathway that regulates ferroptosis through cystine metabolism and is closely linked to chronic kidney disease [39]. Significant decreases in the levels of SLC7A11 and GPX4 were observed in DN patients, DN mouse models, and high glucose-induced HK-2 cells [12, 40]. We discovered that TSN could regulate the SLC7A11/GSH/GPX4 axis, inhibit ferroptosis, and reduce renal tubular injury both in vivo and in vitro. To gain further insight into the mechanisms through which TSN mitigates renal tubular injury, we employed the ferroptosis-specific inducer erastin. Erastin can target and inhibit the molecular switch system Xc-, reduce cystine intake, decrease GSH synthesis, and lower GPX4 activity [41]. After co-incubating erastin and TSN in high-glucose induced HK-2 cells, the study found that erastin antagonized the role of TSN in regulating the SLC7A11/GSH/GPX4 axis to inhibit ferroptosis and attenuate renal tubular injury. This further clarifies the nephroprotection mechanisms of TSN.

## Conclusions

In conclusion, this study has demonstrated that ferroptosis mainly occurs in the renal tubules of DN. The result data of present study also indicated that TSN may decrease tubular injury, reduce urinary microalbumin levels, and protect renal function by inhibiting ferroptosis through the regulation of the SLC7A11/GSH/GPX4 axis.

## Supplementary Information

**Additional file 1.**

**Additional file 2.**

## Data Availability

Data used to support the findings of this study are available from the corresponding author upon request.

## Abbreviations

ACEIs: Angiotensin-converting enzyme inhibitors
ARBs: Angiotensin receptor blockers
BPI: Base peak ion
BUN: Blood urea nitrogen
DN: Diabetic nephropathy
GPX4: Glutathione peroxidase 4
GSH: Glutathione
GSSG: Oxidized glutathione
KIM-1: Kidney injury molecule-1
L-ROS: Lipid-reactive oxygen species
MDA: Malondialdehyde
NGAL: Neutrophil gelatinase-associated lipocalin
RBG: Random blood glucose
SCr: Serum creatinine
SLC7A11: Solute Carrier Family 7, Member 11
TCM: Traditional Chinese medicine
TECs: Tubular epithelial cells
TEM: Transmission electron microscope
TSN: Tangshenning
UAER: Urinary microalbumin excretion rate
UPLC-QTOF/MS: Ultrahigh pressure liquid chromatography-quadrupole-time of flight mass spectrometry
