# Supplementary material for: Suppression of ferroptosis through the SLC7A11/glutathione/glutathione peroxidase 4 axis contributes to the therapeutic action of the Tangshenning formula on diabetic renal tubular injury
Source: Chin Med. 2024 Oct 29;19:151. doi: 10.1186/s13020-024-01007-8 (PMC11523893; doi:10.1186/s13020-024-01007-8)

Figure S1: Base peak ion chromatograms (BPI) of TSN-containing and blank serum. (a) BPI of TSN-containing serum in the positive mode. (b) BPI of blank serum in the positive mode. (c) BPI of TSN-containing serum in the negative mode. (d) BPI of blank serum in the negative mode.

a

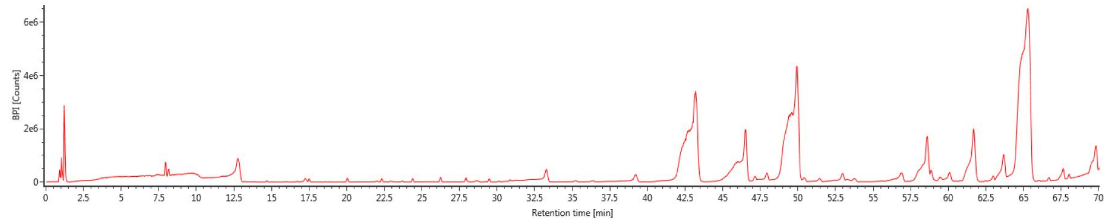

b

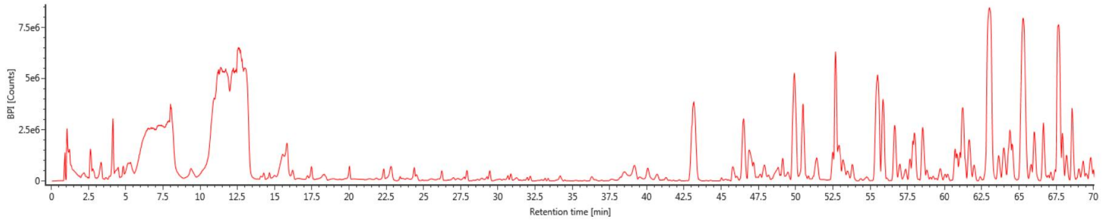

c

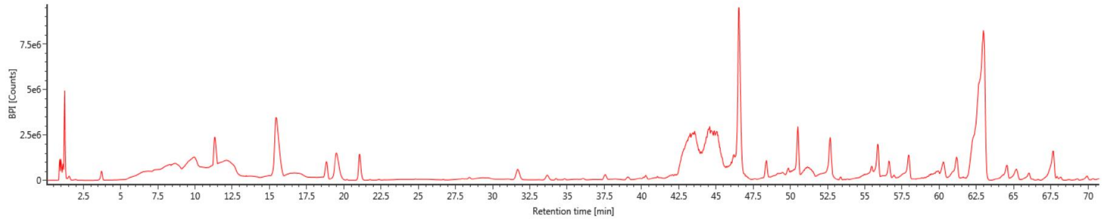

d

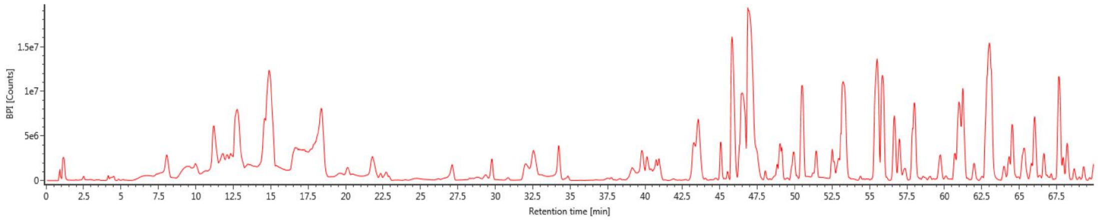

Supplement: Supplementary file 1 — Additional file 1. [file 13020_2024_1007_MOESM1_ESM.pdf]
