# Supplementary material for: Suppression of ferroptosis through the SLC7A11/glutathione/glutathione peroxidase 4 axis contributes to the therapeutic action of the Tangshenning formula on diabetic renal tubular injury
Source: Chin Med. 2024 Oct 29;19:151. doi: 10.1186/s13020-024-01007-8 (PMC11523893; doi:10.1186/s13020-024-01007-8)
Supplement: Supplementary file 2 — Additional file 2. [file 13020_2024_1007_MOESM2_ESM.pdf]

Table S1: Chemical composition of TSN.

NO.	Observed RT (min)	Component name	Formula	Neutral mass (Da)	Mass error (ppm)	ESI mode
1	5.40	Gallic acid	$C_7H_6O_5$	170.02152	3.8	positive
2	24.28	Coumarin	$C_9H_6O_2$	146.03678	4.4	positive
3	27.59	Tetramethylpyrazine	$C_8H_{12}N_2$	136.10005	6.7	positive
4	29.35	Calycosin-7-O-β-D-glucoside	$C_{22}H_{22}O_{10}$	446.12130	1.1	positive
5	33.26	4-hydroxy-3-butylphthalide	$C_{12}H_{14}O_3$	206.09429	2.8	positive
6	36.44	Senkyunolide	$C_{12}H_{16}O_2$	192.11503	2.7	positive
7	37.61	Ononin	$C_{22}H_{22}O_9$	430.12638	1.2	positive
8	40.27	Methylnissolin	$C_{17}H_{16}O_5$	300.09977	3.2	positive
9	40.27	9-O-Methylnissolin 3-O-glucoside	$C_{23}H_{26}O_{10}$	462.15260	1.1	positive
10	42.08	Calycosin	$C_{16}H_{12}O_5$	284.06847	3.8	positive
11	42.19	Isomucronulatol	$C_{17}H_{18}O_5$	302.11542	2.5	positive

12	42.48	Quercetin	$C_{15}H_{10}O_7$	302.04265	3.7	positive
13	52.42	Isoastragaloside IV	$C_{41}H_{68}O_{14}$	784.46091	0.2	positive
14	54.56	Astragaloside VI	$C_{47}H_{78}O_{19}$	946.51373	1.1	positive
15	56.75	Astragaloside III	$C_{41}H_{68}O_{14}$	784.46091	0.6	positive
16	57.24	Astragaloside IV	$C_{41}H_{68}O_{14}$	784.46091	1.1	positive
17	60.77	Astramembrannin II	$C_{35}H_{58}O_9$	622.40808	-0.3	positive
18	60.82	Agroastragaloside Ⅲ	$C_{51}H_{82}O_{21}$	1030.53486	2.1	positive
19	61.18	Astragaloside II	$C_{43}H_{70}O_{15}$	826.47147	0.4	positive
20	62.83	Astragaloside Ⅷ	$C_{47}H_{76}O_{17}$	912.50825	1.7	positive
21	68.29	Sedanolide	$C_{12}H_{18}O_2$	194.13068	3	positive
22	68.32	Linolenic acid	$C_{18}H_{30}O_2$	278.22458	1.3	positive
23	68.86	ligustilide	$C_{12}H_{14}O_2$	190.09938	3.4	positive
24	70.45	Isoastragaloside Ⅰ	$C_{45}H_{72}O_{16}$	868.48204	1	positive

25	79.24	Physcion	$C_{16}H_{12}O_5$	284.06847	3.4	positive
26	8.19	Protocatechuic acid	$C_7H_6O_4$	154.02661	-7.6	negative
27	16.80	Sanguiin H-4	$C_{27}H_{22}O_{18}$	634.08061	2.4	negative
28	16.80	d-catechin	$C_{15}H_{14}O_6$	290.07904	-2.4	negative
29	17.39	caffeic acid	$C_9H_8O_4$	180.04226	-7.7	negative
30	18.75	(-)-epicatechin	$C_{15}H_{14}O_6$	290.07904	-5.6	negative
31	23.45	Riboflavin	$C_{17}H_{20}N_4O_6$	376.13828	-4.5	negative
32	25.17	Ferulic acid	$C_{10}H_{10}O_4$	194.05791	-11.1	negative
33	31.39	Isoquercitrin	$C_{21}H_{20}O_{12}$	464.09548	1.2	negative
34	35.55	Isorhamnetin-3-glucoside	$C_{22}H_{22}O_{12}$	478.11113	2.8	negative
35	41.68	Kaempferide-3-glucoside	$C_{22}H_{22}O_{11}$	462.11621	-1	negative
36	42.20	isomucronulatol 7-O-glucoside	$C_{23}H_{28}O_{10}$	464.16825	-1	negative
37	43.97	Chrysophanol	$C_{15}H_{10}O_4$	254.05791	-4.2	negative

38	48.63	Kaempferol	$C_{15}H_{10}O_6$	286.04774	-3.3	negative
39	49.12	3'-methoxy-5'-hydroxyisoflavone-7-O-β-D-glucoside	$C_{22}H_{22}O_{10}$	446.1213	-3.7	negative
40	50.40	Astragaloside Ⅴ	$C_{47}H_{78}O_{19}$	946.51373	5.3	negative
41	54.50	Aloeemodin	$C_{15}H_{10}O_5$	270.05282	-7.1	negative
42	57.44	Rhein	$C_{15}H_8O_6$	284.03209	-3.7	negative
43	62.98	Agroastragaloside Ⅳ	$C_{49}H_{80}O_{20}$	988.52429	5.1	negative
44	67.18	Acetylastragaloside I	$C_{47}H_{74}O_{17}$	910.49260	4.7	negative
45	68.98	Tormentic acid	$C_{30}H_{48}O_5$	488.35017	-1.9	negative
46	69.52	Emodin	$C_{15}H_{10}O_5$	270.05282	-4	negative
47	72.60	Astragaloside I	$C_{45}H_{72}O_{16}$	868.48204	4.6	negative
48	78.94	Corosolic acid	$C_{30}H_{48}O_4$	472.35526	-1.9	negative
49	87.24	Linolenic acid	$C_{18}H_{30}O_2$	278.22458	-5.4	negative
50	89.79	Mongholicoside Ⅰ	$C_{36}H_{60}O_9$	636.42373	2.1	negative

51	100	Ursolic acid	$C_{30}H_{48}O_3$	456.36035	-0.8	negative
